# Predictors of glycemic control among patients with Type 2 diabetes: A longitudinal study

**DOI:** 10.1186/1471-2458-5-36

**Published:** 2005-04-17

**Authors:** Stephen R Benoit, Regina Fleming, Athena Philis-Tsimikas, Ming Ji

**Affiliations:** 1 University of California, San Diego – San Diego State University Preventive Medicine Residency, Dept. of Family and Preventive Medicine, MC-0811, 9500 Gilman Drive, La Jolla, CA 92093-0811, USA; 2 The Whittier Institute for Diabetes, Scripps Health, 9894 Genesee Ave., La Jolla, CA 92037, USA; 3 San Diego State Graduate School of Public Health, 5500 Campanile Drive, San Diego, CA 92182-4162, USA

## Abstract

**Background:**

Diabetes is the sixth leading cause of death and results in significant morbidity. The purpose of this study is to determine what demographic, health status, treatment, access/quality of care, and behavioral factors are associated with poor glycemic control in a Type 2 diabetic, low-income, minority, San Diego population.

**Methods:**

Longitudinal observational data was collected on patients with Type 2 diabetes from Project Dulce, a program in San Diego County designed to care for an underserved diabetic population. The study sample included 573 patients with a racial/ethnic mix of 53% Hispanic, 7% black, 18% Asian, 20% white, and 2% other. We utilized mixed effects models to determine the factors associated with poor glycemic control using hemoglobin A1C (A1C) as the outcome of interest. A multi-step model building process was used resulting in a final parsimonious model with main effects and interaction terms.

**Results:**

Patients had a mean age of 55 years, 69% were female, the mean duration of diabetes was 7.1 years, 31% were treated with insulin, and 57% were obese. American Diabetes Association (ADA) recommendations for blood pressure and total cholesterol were met by 71% and 68%, respectively. Results of the mixed effects model showed that patients who were uninsured, had diabetes for a longer period of time, used insulin or multiple oral agents, or had high cholesterol had higher A1C values over time indicating poorer glycemic control. The younger subjects also had poorer control.

**Conclusion:**

This study provides factors that predict glycemic control in a specific low-income, multiethnic, Type 2 diabetic population. With this information, subgroups with high risk of disease morbidity were identified. Barriers that prevent these patients from meeting their goals must be explored to improve health outcomes.

## Background

Approximately 13 million people have been diagnosed with diabetes in the United States and an additional 5.2 million do not yet know they have the disease [1]. Of these people, 90–95% have Type 2 diabetes [2]. Diabetes has been among the top ten leading causes of death in the United States since 1932 and is now the sixth leading cause of death [3]. Because of the magnitude of the burden of disease, the Healthy People 2010 objectives include goals of reducing diabetes-related deaths and increasing the monitoring frequency of glucose control and chronic complications [4]. The United Kingdom Prospective Diabetes Study and Kumamoto study confirm that improved glucose control reduces the microvascular complications in Type 2 diabetes such as retinopathy, nephropathy, and neuropathy [5,6]. Because of these findings, new standards of care and new models of health care delivery have emerged [7].

Disease management programs that incorporate group patient education, nutrition consultation, case management as well as close clinical care have been effective [8]. Project Dulce, an initiative of Community Health Improvement Partners, the Council of Community Clinics, and The Whittier Institute for Diabetes was started in 1998 in San Diego County. The program has a multifaceted approach and focuses on providing care to minority groups that often lack access to medical services.

Although it is known that improved glycemic control improves microvascular outcomes, less is known about the factors that influence control. Harris et al. [9] examined racial and ethnic differences in glycemic control in patients with Type 2 diabetes using the Third National Health and Nutrition Examination Survey (NHANES III) and found that black women, Mexican-American men, those treated with insulin or oral antiglycemic medications, and patients over 60 years of age had poorer glycemic control. Shorr et al. [10] studied the relationship between age and glycemic control and found no significant differences between age groups. Nichols et al. [11] found that age, body mass index (BMI) and emotional distress were significantly related to glycemic control in a health maintenance organization population in Oregon. Blaum et al. [12] found that disease duration, C peptide levels, poor self-care, and failure to receive diet recommendations were related to control in a mostly white, primary care population in Michigan. Project Dulce, however, is a distinct population of low-income, multiethnic patients with a high proportion of Hispanics and Asians. Therefore, it is useful to study patient characteristics associated with glycemic control in this unique setting. Prior studies have not accounted for fluctuations in glycemic control over time. We used a longitudinal data analysis approach to account for glycemic variation and thus maximized the amount of information that can be drawn from the data.

## Methods

### Data study sample

Project Dulce is a nurse-based diabetes disease management system in San Diego, California [13]. Patients with diabetes are referred to Project Dulce by primary care providers. Once the patient is referred, the nurse educator conducts an initial assessment and follows the American Diabetes Association (ADA) standards of appropriate physical and laboratory exams and referrals to specialists (7). Hemoglobin A1C (A1C) is monitored quarterly and lipids, urine microalbumin, thyroid stimulating hormone (TSH), and retinal exams are completed yearly or more frequently as needed. At each visit, height, weight, blood pressure, foot exams, and glucometer results are reviewed.

The nurse educator is the case manager and follows-up on missed patient appointments and identifies individual service and access needs of his/her panel of patients. The nurse also communicates with the primary care physician regarding clinical care issues. Project Dulce Dieticians see patients referred by the nurse educators. The program is active in seventeen sites including community clinics and hospital ambulatory care centers throughout San Diego County. Project Dulce uses the same procedures and supervision at each site and tracks patients with the Diabetes Electronic Management System (DEMS) software. The database contains demographic, health status, treatment, laboratory, and behavioral factors for each patient and collects the information over time. This study included data from July 18, 2000 to October 7, 2002 and was approved by the Institutional Review Board of San Diego State University.

### Eligibility criteria

For purposes of this analysis, we selected patients with Type 2 diabetes, reducing the population size from 1,728 to 1,357. To avoid bias and ensure that the study population was actively participating in the Project Dulce program, inclusion criteria were established. The patient required: 1) at least two A1C values at least six months apart, 2) participation in the program for at least six months, and 3) at least three Project Dulce provider visits. Of the 1,357 Type 2 diabetes patients in the database, 573 met these criteria.

### Measures

#### a. Demographics

Demographic variables included gender, age, race/ethnicity, and primary language. All were of low socioeconomic status. For purposes of this study, five racial/ethnic categories were created: Hispanic, Asian (including Indian), black, white, and other.

#### b. Glycemic control

A1C is a laboratory value that indicates glycemic control over a 2 to 3 month period; values less than 7% are considered optimal. A1C was our outcome of interest and was evaluated over time by examining the patients' A1C laboratory results over a 24 month period. Since Project Dulce follows the ADA recommendations of checking A1C values every 3 months, values were placed in 3 month block intervals, using the patients' initial provider visit as the reference starting point. A1C laboratory data on individual patients was not always precisely three months apart so approximations were necessary. Since A1C is an indicator of glycemic control over a 2 to 3 month period, we used a plus or minus 1.5 month approximation. For example, a three-month lab was considered an A1C measurement 1.5 to 4.5 months after the initial Project Dulce visit. A baseline A1C value was considered a measurement between 2.8 months before the initial Project Dulce visit to 1.5 months after the visit. Since a two year time period was of interest in this study, only A1C values falling within 2.8 months of the initial visit or 25.5 months after this visit were included in the analysis. If more than one A1C value was available in a particular 3 month time block, the first measurement within that block was used.

#### c. Diabetes severity

The difference in dates of the patient's initial Project Dulce visit and the diabetes diagnosis date estimated disease duration in years. Medicines used for glucose control (insulin, sulfonylureas, metformin, glitazones, alpha glucosidase inhibitors, meglitinides) were categorized into three levels: 1) insulin alone or insulin with oral agents, 2) more than one oral agent but no insulin, and 3) one oral medication or no medication at all. Since a patient's pharmacotherapy changed over time, we created a coding strategy. If the patient used insulin at any point over the two year study period, he/she was placed in the insulin category. Similarly, if the patient ever used more than one oral medication but never used insulin, the patient was placed in the more than one oral agent category.

#### d. Health status

Clinical characteristics considered included systolic (SBP) and diastolic (DBP) blood pressure, total and HDL cholesterol, urine microalbumin-to-creatinine ratio, and BMI. Mean values of the clinical variables were used over the appropriate time period. In univariate analysis, cutpoints were created based on ADA guidelines [7]. Hypertension was considered SBP or DBP greater than or equal to 130 mm Hg and 80 mm Hg, respectively. Total cholesterol or HDL greater than equal to 200 mg/dl and 45 mg/dl, respectively were defined as dyslipidemia. Urine microalbumin-to-creatinine levels between 30 and 299 ug/mg was considered microalbuminuria and greater than or equal to 300 ug/mg was considered clinical albuminuria. BMI greater than or equal to 30 kg/m^2 ^was considered obese.

#### e. Access / quality of care

Most of the patients in Project Dulce have County Medical Services, an insurance program funded by San Diego County to care for the medically indigent adult population (MIA). The remainder are uninsured and pay out-of-pocket to enroll in the program or are covered by Medicare, Medicaid, or private insurance. For purposes of this study insurance status was categorized as uninsured, MIA, or insured (insured = Medicare, Medicaid or private insurance). The number of provider visits, duration in the program, and whether the patient was seen by a Project Dulce nutritionist was also recorded.

#### f. Behavioral factors

Behavioral factors in the model included smoking and the number of Project Dulce diabetes education classes attended.

### Descriptive and Univariate analysis

The number and proportion of patients were recorded for each variable within demographic, diabetes severity, health status, access/quality of care, and behavioral factor groups. In addition, mean A1C values were compared across levels of each variable. Univariate analysis using a t-test or One-Way ANOVA was used to assess significant differences in mean A1C. If significant differences were found in ANOVA, the Duncan function in SAS 8.1 was used to asses individual differences.

### Mixed effects model

Mixed effects models were used to assess glycemic control by analyzing the repeated measure data of A1C values. The A1C values were skewed and therefore log transformed in order to meet the normal distribution assumption. Several correlation structures including Compound Symmetry, Unstructured, First-order Autoregressive, and Toeplitz were assessed for each model. We used Akaike's Information Criterion (AIC) to select the appropriate correlation structure [14]. All model fittings were implemented using SAS PROC MIXED and the model with the smallest AIC was considered the best fit [15].

Univariate associations were performed to assess the best functional form of the variables. Continuous variables were assessed as linear and curve-linear with the addition of quadratic terms. Using a hierarchical model building process, clusters of variables were added in, one-by-one. All models included baseline A1C and time (in months) since these two variables were considered essential to control for in assessing glycemic control in the longitudinal format. In each model, the AIC of the best-fitted correlation structure was noted.

All variables significant in one of the hierarchical models at an alpha level of 0.15 were placed together in a separate model. Finally, a parsimonious mean effects model was created, leaving only variables significant at the alpha level of 0.05. Variable by time interaction terms were entered into the parsimonious mean effects model in a clustered process. Significant interaction terms at the alpha level of 0.05 were then placed together with the parsimonious model. Once the model variables were finalized, correlation structures for fixed and random effects were verified.

## Results

Table 1 shows the study population characteristics and univariate associations of factors with glycemic control.

**Table 1:** Population characteristics and univariate associations of factors with mean A1C. Project Dulce, 2000–2002 (N = 573)

	n (%)	Mean A1C (%)	p value
**Demographic factors**			
Gender, n (%)			0.99
1. Female	392 (68.7%)	7.63	
2. Male	179 (31.4%)	7.63	
			
Age, n (%)	Mean = 55.4 ± 10.1		< 0.0001
1. < 50 years	149 (26.0%)	7.89	1 > 2, 3
2. 50–65	355 (62.0%)	7.57	
3. > 65	69 (12.0%)	7.36	
			
Ethnicity, n (%)			< 0.0001
1. Hispanic	304 (53.3%)	7.84	2 > 3, 4
2. Black	39 (6.8%)	7.96	4 < 2
3. Asian	100 (17.5%)	7.06	3 < 1,2, 5
4. Other	11 (1.9%)	7.46	
5. White	116 (20.4%)	7.55	
			
Primary Language, n (%)			0.18
1. Not English	302 (52.8%)	7.58	
2. English	270 (47.2%)	7.68	
			
**Diabetes severity**			
Diabetes duration, n (%)	Mean = 7.1 ± 7.1		< 0.0001
1. < 1 year	122 (21.9%)	7.04	4 > 3 > 2 > 1
2. 1 – 5 years	195 (34.9%)	7.45	
3. 6 – 10 years	93 (16.7%)	7.77	
4. > 10 years	148 (26.5%)	8.19	
			
Medicine, n (%)			< 0.0001
1. Insulin alone or insulin + oral agents	177 (30.9%)	8.32	1 > 2 > 3
2. > 1 oral agent (no insulin)	284 (49.6%)	7.58	
3. No medicine or 1 oral agent	112 (19.5%)	6.47	
			
**Health status**			
Systolic blood pressure, n (%)	Mean = 125.2 ± 11.9		0.33
1. < 130 mm Hg	404 (70.5%)	7.65	
2. ≥ 130 mm Hg	169 (29.5%)	7.57	
			
Diastolic blood pressure, n (%)	Mean = 72.1 ± 6.6		0.19
1. < 80 mm Hg	506 (88.3%)	7.64	
2. ≥ 80 mm Hg	67 (11.7%)	7.48	
			
Total cholesterol, n (%)	Mean = 187.6 ± 36.0		< 0.0001
1. < 200 mg/dl	386 (68.2%)	7.45	
2. ≥ 200 mg/dl	180 (31.8%)	8.03	
			
HDL, n (%)	Mean = 45.3 ± 12.0		0.27
1. ≤ 45 mg/dl	322 (56.6%)	7.73	
2. > 45 mg/dl	247 (43.4%)	7.59	
			
Urine Microalbumin / creatinine, n (%)	Mean = 139.9 ± 480.7		< 0.0001
1. < 30 ug/mg	356 (63.6%)	7.40	3 > 2 > 1
2. 30 – 299 ug/mg	160 (28.6%)	7.84	
3. ≥ 300 ug/mg	44 (7.8%)	8.55	
			
Body Mass Index, n (%)	Mean = 32.5 ± 7.5		0.003
1. < 30 kg/m^2^	246 (43.2%)	7.50	
2. ≥ 30 kg/m^2^	323 (56.8%)	7.72	
			
**Access/quality of care**			
Insurance, n (%)			< 0.0001
1. Uninsured	169 (29.5%)	8.10	1 > 2, 3
2. County Medical Services	249 (43.5%)	7.39	
3. Insurance	155 (27.0%)	7.50	
			
Number of provider visits, n (%)	Mean = 10.2 ± 4.4		0.002
1. 3 – 6	118 (20.6%)	7.72	4 > 2, 3
2. 7 – 10	225 (39.3%)	7.56	
3. 11 – 15	166 (29.0%)	7.51	
4. > 15	64 (11.1%)	7.93	
			
Duration in program, n (%)	Mean = 15.7 ± 5.5		0.0001
1. 6 – 12 months	212 (37%)	7.88	
2. > 12 – 24 months	361 (63%)	7.53	
			
Seen by nutritionist, n (%)			0.32
1. Yes	475 (82.9%)	7.61	
2. No	98 (17.1%)	7.71	
			
**Behavioral Factors**			
Smoking habit, n (%)			0.29
1. Current	63 (12.5%)	7.58	
2. Past	162 (32.0%)	7.60	
3. Never	281 (55.5%)	7.72	
			
Diabetes Classes Attended, n (%)	Mean = 1.5 ± 3.0		0.002
1. 0	358 (74.3%)	7.60	2 > 1 > 3
2. 1–4	42 (8.7%)	7.88	
3. 5 +	82 (17.0%)	7.31	

Multiple comparison tests in ANOVA were done with the Duncan function in SAS 8.1

### a. Demographics

There were more females (68.7%) than males (31.3%). The mean age was 55.4 years and the younger group had a higher mean A1C (7.9%) than the other two age groups. Hispanics represented 53.3% of the study sample and Asians had lower mean A1C values (7.1%) than Hispanics (7.8%), blacks (8.0%), and whites (7.6%). The majority of the patients (52.8%) used a language other than English as their primary language.

### b. Diabetes severity

The mean duration of diabetes was 7.1 years and increasing duration of disease resulted in progressively higher mean A1C values. Insulin users comprised 30.9% of the study population and had higher mean A1C values (8.3%) than multiple oral medication users (7.6%) and those on one oral agent or no medication (6.5%).

### c. Health status

Mean systolic and diastolic blood pressures were within ADA target recommendations (less than 130 and 80 mm Hg, respectively) with 70.5% and 88.3% of the study population, respectively, meeting the goals. Mean total cholesterol was 187.6 mg/dl and those with lower total cholesterol (less than 200 mg/dl) had lower mean A1C values (7.5%) than those with higher total cholesterol levels (8.0%). Patients with clinical albuminuria comprised 7.8% of the study population and had higher mean A1C values (8.6%) than those with microalbuminuria (7.8%) and those with no microalbuminuria (7.4%). The majority (56.8%) of the patients were obese and they had higher mean A1C values (7.7%) than those who were not obese (7.5%).

### d. Access / quality of care

The largest (43.5%) group of patients were enrolled in San Diego County Medical Services followed by the uninsured (29.5%). Uninsured patients had higher mean A1C values (8.1%) than those with insurance (7.5%) or County Medical Services (7.4%). Most (63.0%) of the study patients were enrolled in Project Dulce over one year and this group had lower mean A1C values (7.5%) compared to the group enrolled for one year or less (7.9%). Patients with greater than 15 provider visits had higher mean A1C values (7.9%) than those with less provider visits. The majority (82.9%) of the patients had seen a Project Dulce nutritionist.

### e. Behavioral factors

Current smokers comprised 12.5% of the study population. Patients who attended 5 or more Project Dulce diabetes classes had lower (7.3) mean A1C values than those who attended no classes (7.6) and those attending 1 to 4 classes (7.9).

Table 2 provides the regression results for the multivariate mixed effects model analysis which illustrates the factors associated with glycemic control. Insurance status, disease duration, pharmacotherapy, and cholesterol level were significantly associated with glucose control. Fluctuation in mean A1C over time also differed by age (age*month). Using the insured as the reference group, the uninsured had a 5.2% higher A1C level. Patients who had diabetes over ten years had a 15.3% higher A1C level compared to those who had diabetes less than one year. Similarly, patients who had diabetes six to ten years and one to five years had significantly higher A1C values compared to those with diabetes less than one year. Patients who required insulin had a 22.4% higher A1C and those who required more than one oral medication had a 12.0% higher A1C compared to hose who used one oral medication or no medication at all. On average, for every 0.65 mmol/l (25 mg/dl) increase in total cholesterol, the A1C value was 2.6% higher.

**Table 2:** Multivariate mixed effects model to assess characteristics associated with glycemic control. Project Dulce, 2000–2002 (N = 555)

	Estimate	p value	Translation*
Baseline A1C	0.06050	<0.0001	
Month (0 – 24)		<0.0001	
Insurance		0.003	
Uninsured	0.02198	0.06	5.2% increase in A1C ^1^
County Medical Services (MIA)	-0.01300	0.20	
Insured (ref)	-	-	
Diabetes duration		<0.0001	
> 10 years	0.06169	<0.0001	15.3% increase in A1C
6 – 10 years	0.03555	0.008	8.5% increase in A1C
1 – 5.9 years	0.03253	0.003	7.8% increase in A1C
< 1 year (ref)	-	-	
Medicine		<0.0001	
Insulin alone or insulin + oral agents	0.08768	<0.0001	22.4% increase in A1C
> 1 oral agent (no insulin)	0.04930	<0.0001	12.0% increase in A1C
No medicine or 1 oral agent (ref)	-	-	
Total cholesterol (0.65 mmol/l (25 mg/dl) interval)	0.01115	<0.001	2.6% increase in A1C
Age * month		<0.001	

* Formula for calculating change in A1C = 10^(estimate) ^- 1

^1^10^(0.02198) ^- 1 = 0.052

**Figure 1:**
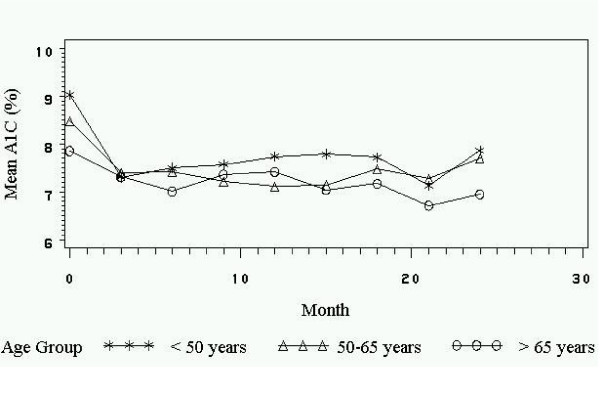
Fluctuation in mean A1C values over time by age group. Project Dulce, 2000–2002

How mean A1C fluctuated over time differently for various age groups is best interpreted with a plot. Although age was a continuous variable in this analysis, for purposes of interpretation, we created three categories. Figure 1 demonstrates that the less than 50 years of age group initially declined in A1C from baseline to three months but then slowly rose during the next fifteen months. The two older age groups had lower mean A1C values that fluctuated during those fifteen months.

## Conclusion

Univariate analysis indicates that multiple variables are associated with glycemic control. Age, race/ethnicity, disease duration, medication, number of Project Dulce visits, duration in Project Dulce, total cholesterol, microalbumin-to-creatinine ratio, BMI, insurance status, and the number of diabetes classes attended were all significant. However, after controlling for baseline A1C, time and other demographic, disease severity, health status, and access/quality of care factors, only age, insurance status, disease duration, pharmacotherapy, and total cholesterol were significant in the final model with main effects or two-way interaction terms.

The association of insurance status with glycemic control contradicts previous studies. Harris et al. [9] did not find an association between glycemic control and insurance coverage or socioeconomic status using NHANES III data, a representative sample of the U.S. population. Similarly, the Michigan community study [12], a study of blacks and whites in South Carolina [16], and a study of whites and Mexican-Americans in Texas [17] did not find an association between glycemic control and socioeconomic status. Our population, however, is distinct. It is a multiracial/ethnic population and all have a low socioeconomic status. Within this low-income population, the uninsured had poorer glycemic control. They represent a subgroup of patients that struggle to find care for a disease that requires close monitoring. Their disease state may have been out-of-control before entering Project Dulce and thus more difficult to gain control. They may also have lacked self-care skills or basic knowledge of diabetes since their care in the past was likely sporadic. Perhaps, factors such as dietary practices, physical exercise, and education level were important predictors and differed in this subgroup [18,19]. Providers may have difficulty procuring medication and equipment for this group of patients. All of these factors could help explain the discrepancy but were not accounted for in this study.

Study findings have differed on the association of glycemic control and disease duration. Similar to Blaum et al. [12] and contradictory to Nichols et al. [11], we found that the longer someone had been diagnosed with diabetes, the harder it was to maintain glycemic control. Although self-care skills could improve with longer duration of disease, resistance to medication and the need for higher doses or additional medications increase over time. Insulin use is also a factor of disease severity and was a predictor of poorer glycemic control in this study. The mean A1C value (8.3%) of insulin users in our study was equivalent to the mean value of insulin users in the NHANES III data [9]. They also found insulin users to have poorer glucose control.

Among health status factors, high total cholesterol was associated with poorer glycemic control. Since patients with diabetes are already at high risk for cardiovascular disease, this finding reinforces the need to aggressively screen and treat elevated cholesterol.

Although other health status factors were not associated with glycemic control in multivariate analysis, it is important to assess the health status of Project Dulce patients compared to other populations. Harris [20] studied health status and outcomes using NHANES III. Blood pressure was elevated (greater than or equal to 140/90 mm Hg) in 55% to 65% of the population compared to Project Dulce's 30% for SBP (greater than 130 mm Hg) and 12% for DBP (greater than 80 mm Hg). Total cholesterol was greater than or equal to 200 mg/dl in 62% to 69% of the NHANES III population compared to 32% of the Project Dulce population. Cigarette smokers compreised 18% to 24% of NHANES III and 13% of Project Dulce. The prevalence of microalbuminuria and obesity, however, was higher in the Project Dulce population than NHANES III, 36.4% vs. 26% to 30% and 57% vs. 34% to 54%, respectively. The fact that a higher proportion of Project Dulce patients compared to a representative sample of the U.S. population were meeting ADA blood pressure and cholesterol recommendations suggests the positive impact and importance of community disease management programs in low-income, multiracial/ethnic communities.

Prior studies have demonstrated race/ethnicity as a predictor of glycemic control with higher proportions of poorly controlled patients among black women and Mexican-American men [9]. In univariate analysis, our study found that Asians had better glycemic control than Hispanics, blacks, and whites. However, this relationship disappeared in multivariate analysis after taking other factors into account. Perhaps the reason why our finding differs from other studies is that regardless of race/ethnicity, all study patients were of low socioeconomic status. Prior studies examined race/ethnicity in populations with differing socioeconomic status levels.

Similar to results from Shorr et al.'s study [10] using NHANES III data, our study found that in multivariate analysis, age was not a significant main effect in predicting glucose control. However, the significant age by time interaction term (age*month) indicates that A1C patterns over time differed between age groups. Figure 1 shows that while the 50 to 65 and 65 and over age groups' A1C values fluctuated over time, the younger age group's A1C values steadily rose. Nichols et al. [11] also found poorer metabolic control among the younger age group. Since our longitudinal analysis accounted for fluctuations in A1C values, we were able to study A1C pattern differences. It would be interesting to see if this same A1C pattern difference among age groups exists in a representative sample of the U.S. population.

The strength of the current study was the use of mixed effects models. This is the first study that used a longitudinal approach to find factors associated with glycemic control. Incorporating repeated measures over time accounts for fluctuations in glucose control and maximizes the amount of information that can be drawn from the data. Another advantage was the size and diversity of the population which included large numbers of Hispanic, Asian, and white patients, far more diverse than studies using representative samples of the U.S. population.

While the race/ethnic population was diverse, the socioeconomic status of the population was not. Most of the patients were of very low income which limits the generalizability of the study results. Missing data was also a limitation. Missing quarterly A1C values was common but mixed effects models still yield unbiased estimates provided that the missing data was missing at random (MAR) [14].

Finally, multiple factors affect glycemic control. The mixed effects model incorporated demographic, disease severity, health status, access/quality of care, and behavioral factors but these are just some of the possible factors that affect glycemic control. Psychological and biological factors, self-care skills, knowledge of disease and education level, diet, exercise, other comorbid diseases, etc. were not explained by this model. Nichols et al.'s [11] study found that only 9% of the variability in glycemic control was explained by the factors in their model and suggested that personal characteristics may not explain a lot of differences in glycemic control among patients with Type 2 diabetes.

This study identified patients with poorer glycemic control in Project Dulce. The findings should not be generalized to all patients with Type 2 diabetes but can be applied to racial/ethnically diverse, low-income populations. Those who were uninsured, had diabetes for a longer period of time, used insulin or multiple oral agents, or had high cholesterol had poorer glycemic control. The younger population also lagged behind others. Secondarily, this study showed that a high proportion of the patients were meeting ADA's blood pressure and cholesterol recommendations, suggesting that community disease management programs in low-income populations can be effective and may contribute to improved health outcomes.

This study provides a useful methodology to assess disease management systems that collect longitudinal data. It does not provide answers to why patients are not optimally controlled but does provide a starting point from which to investigate and address the obstacles that prevent patients with diabetes from reaching their metabolic targets.

## Competing interests

The author(s) declare that they have no competing interests.

## Authors' contributions

SB designed and wrote the study and analyzed the data. MJ participated with the design, analysis and interpretation of the data and revision of the paper. RF participated in the design and writing of the paper. AT acquired the data and participated in the design and revision of the paper. All authors read and approved the final manuscript.

## Pre-publication history

The pre-publication history for this paper can be accessed here:


